# Ischemia–Reperfusion Intervention: From Enhancements in Exercise Performance to Accelerated Performance Recovery—A Systematic Review and Meta-Analysis

**DOI:** 10.3390/ijerph17218161

**Published:** 2020-11-04

**Authors:** Rhaí André Arriel, Jéssica Ferreira Rodrigues, Hiago Leandro Rodrigues de Souza, Anderson Meireles, Luís Filipe Moutinho Leitão, Antonio Crisafulli, Moacir Marocolo

**Affiliations:** 1 Department of Physiology, Federal University of Juiz de Fora, Juiz de Fora 36036-330, Brazil; rhaiarriel@bol.com.br (R.A.A.); hlrsouza@gmail.com (H.L.R.d.S.); meireles726@gmail.com (A.M.); 2 Department of Agrarian Sciences, Federal Institute of Minas Gerais, Bambuí 38900-000, Brazil; jessica.rodrigues@ifmg.edu.br; 3 Superior School of Education, Polytechnic Institute of Setubal, 2910-761 Setubal, Portugal; luis.leitao@ese.ips.pt; 4 Life Quality Research Centre, 2040-413 Rio Maior, Portugal; 5 Sports Physiology Lab., Department Medical Sciences and Public Health, University of Cagliari, 09124 Cagliari, Italy; crisafulli@tiscali.it

**Keywords:** intermittent occlusion, blood flow occlusion, sports, ergogenic, ischemic postconditioning

## Abstract

It has been demonstrated that brief cycles of ischemia followed by reperfusion (IR) applied before exercise can improve performance and, IR intervention, applied immediately after exercise (post-exercise ischemic conditioning—PEIC) exerts a potential ergogenic effect to accelerate recovery. Thus, the purpose of this systematic review with meta-analysis was to identify the effects of PEIC on exercise performance, recovery and the responses of associated physiological parameters, such as creatine kinase, perceived recovery and muscle soreness, over 24 h after its application. From 3281 studies, six involving 106 subjects fulfilled the inclusion criteria. Compared to sham (cuff administration with low pressure) and control interventions (no cuff administration), PEIC led to faster performance recovery (*p* = 0.004; ES = −0.49) and lower increase in creatine kinase (*p* < 0.001; effect size (ES) = −0.74) and muscle soreness (*p* < 0.001; ES = −0.88) over 24 h. The effectiveness of this intervention is more pronounced in subjects with low/moderate fitness level and at least a total time of 10 min of ischemia (e.g., two cycles of 5 min) is necessary to promote positive effects.

## 1. Introduction

High-level sports performance is dependent on several factors that require high mechanical [1], psychological [2] and physiological [3] demands. Elite competitors are usually submitted to successive high volume and intensity training sessions and/or to multi-days competitions, with short intervals of recovery. These events can lead to physiological [3] and psychological [4] alterations, impairing sports performance. Thus, to increase the resistance to fatigue and to improve performance, many athletes and coaches search post-exercise recovery strategies [5].

In this context, cycles of ischemia–reperfusion (IR) performed immediately after exercise (post-exercise ischemic conditioning—PEIC) are an interesting ergogenic aid to accelerate recovery during high intensity exercise sessions [6,7]. This intervention is actually low cost, non-invasive, easy and quick-to-apply compared to other methods, such as cold-water immersion [8,9]. The IR requires the use of a cuff (tourniquet) on the proximal regions of the lower or upper limbs and the execution of repeated bouts of ischemia interspersed with reperfusion periods [10]. Analyzing the studies with IR and exercise performance, it is possible to verify that the most common applied IR protocols encompass three or four bouts of 5 min of ischemia followed by 5 min of reperfusion among bouts [11,12].

IR interventions were initially used before a prolonged ischemic insult that causes myocardial necrosis. This type of intervention, termed ischemic preconditioning, was able to confer cardiac protection against myocardial infarct [13] and it was associated with low increases in tissue necrosis biomarkers (i.e., creatine kinase (CK)) [14] and improved cardiac performance during exercise [15,16]. Afterwards, the use of IR interventions after prolonged ischemic insult, termed ischemic postconditioning, also promoted protection to heart tissue [14] and reduced oxidative stress [17]. When IR interventions were applied to improve exercise performance, the history was similar: firstly, performance before physical exercise promoted a better skeletal muscle capacity [18], improved performance of swimmers [19], runners [20], and cyclists [21]. Then, application immediately during recovery phase from exercise prevented the drops in performance 24 h, 48 h, and 72 h after an exercise-induced muscle damage, mitigating increases in CK [7]. It is important to note that both the mentioned tissue biomarkers and oxidative stress are associated with drop in sports performance [22,23]. Therefore, during recovery, PEIC application could attenuate tissue and oxidative injury caused by exercise.

The first study that evaluated the PEIC effect during recovery phase [24] employed the intervention immediately after an exercise protocol that involved jumps and repeated sprints. After PEIC, the participants repeated the exercise protocol, and again 24 h later. Beneficial effects were observed both immediately and 24 h post intervention. Specifically, recovery of power production and sprint performance were improved. Compared with other IR studies [25,26], the authors used a short protocol (two bouts of 3 min of ischemia followed by 3 min of reperfusion; 2 cycles × 3 min). Based on this protocol, Northey et al. [27] evaluated the velocity of recovery applying the PEIC immediately after a fatiguing resistance exercise protocol. PEIC was not able to attenuate the loss in muscle force during jumps and the torque during concentric isokinetic contractions 1 h and 24 h later. Similar lack of beneficial results using the same IR protocol were observed in academy rugby players 2 h and 24 h after PEIC application [28] but longer protocols (e.g., three cycles × 5 min) were also efficient to prevent decrements in maximal voluntary contraction, jumps and sprints performance [7,29].

Beyond a limited amount of investigations and controversial results, it is important to describe the heterogeneity of PEIC and exercise protocols to induce fatigue (specific and non-specific), as well as the fitness level of evaluated volunteers. Therefore, it would be appropriate to evaluate the current status and the future perspective of PEIC on physical performance recovery including their possible mechanisms. To this aim, we conducted this review and meta-analysis.

## 2. Materials and Methods

This systematic review with meta-analysis followed the same structure as research articles and conform to the Preferred Reporting Items for Systematic Reviews and Meta-analysis (PRISMA) guidelines.

### 2.1. Database Search

Two independent reviewers, using PubMed, Scopus, SPORTDiscus and Web of Science database, identified potential studies published after January 2012. After conducting a search in the database, it was identified that in 2012 the first study evaluating the PEIC effect on performance recovery was published [24] with the following key terms (i) “intermittent occlusion”; (ii) “ischemic conditioning”; (iii) “ischemic postconditioning”; (iv) “vascular occlusion” combined with “recovery” and “performance recovery”. Only original research studies written in English were included. The literature search was completed on 30 June 2020. Potential studies were selected based on strict criteria including: (i) original research studies; (ii) PEIC performed during post exercise; (iii) evaluation of healthy participants; (iv) completion of an exercise or effort test; (v) analysis of performance recovery. Studies with animal model or non-healthy subjects’ articles, systematic reviews and meta-analysis were excluded. Restrictions such as participant age and fitness level were not applied. 

### 2.2. Study Selection and Quality Assessment

**Figure 1:**
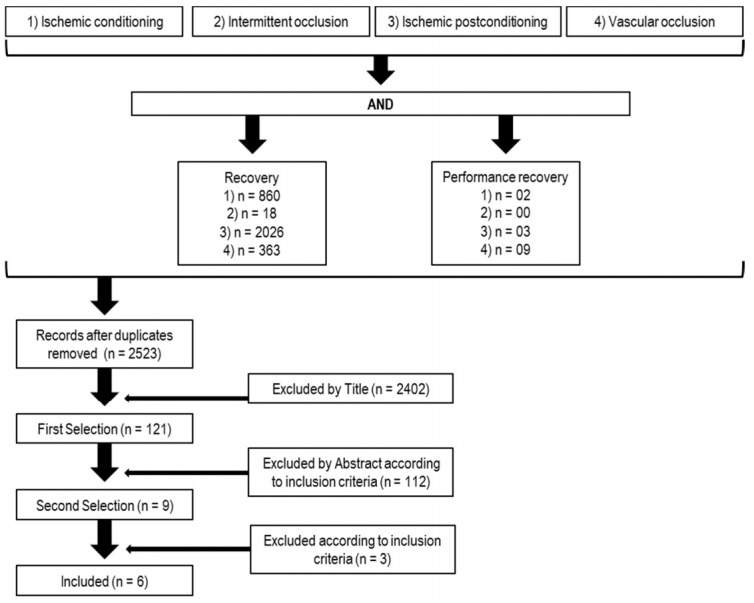
The procedure to select/inclusion of the studies.

All articles were selected that met the criteria for inclusion and evaluation indicated above. The search revealed a total of 3281 articles. Primarily, the duplicates were removed and then the remaining studies were selected by title. If they matched our inclusion criteria, abstracts were checked. Finally, each study which appeared to respect the criteria of eligibility was reviewed (Figure 1).

To estimate the quality of all articles included in this review, a checklist was used (Table 1) based on our previous experience [12,30]. Two investigators analyzed the methodological quality of the articles assigning among three possible scores (Yes = 1 point, Unclear = 1/2 point, No = 0 points) for each item (total of 15) of the checklist. The maximum score was 15 points. The sum of the 15 criteria scores achieved by each paper was used to attribute its general quality. The conclusion of the total scores was taken in agreement with both the investigators. All data collection was conducted by two evaluators separately. Whenever discrepancies were detected, a third party was also involved in the evaluation.

### 2.3. Data Analysis

Each study was read, and data extracted. Descriptive information (e.g., sample size, age, training level and PEIC sets), performance, perceived recovery (PRS), muscle soreness (MS), and CK data were collected from articles or requested to authors via e-mail. When the data were shown only by figures, the webplotdigitizer 4.3 software (apps.automeris.io/wpd) was used to extract them. Within-group change of these variables was determined by calculation of the difference between pre- and post-interventions. The mean relative percentage change was determined by post- minus pre-interventions values divided by pre-intervention value multiplied by 100. To compare the relative percentage change of performance between PEIC and sham/control groups, the Shapiro–Wilk test was performed to verify the normality of data followed by Mann–Whitney test. The GraphPad^®^ (Prism 6.0, San Diego, CA, USA) was used for this analysis.

Only variables assessed in more than three studies were taken into account for the meta-analysis. Therefore, the meta-analysis was conducted separately for performance, CK and MS variables using the Review Manager Software, version 5.4, Copenhagen (Denmark). The level of significance adopted was *p* ≤ 0.05. The effect size (ES) was calculated for each variable using pre-intervention and post-intervention and standard deviations (SDs) values to determine the meaningfulness of the difference. A value <0.2 was considered trivial, >0.2–0.6 small, >0.6–1.2 moderate, >1.2–2.0 large, and >2.0–4.0 very large effect [31]. When a study was lacking in necessary data, the following Equation (1) was used to estimate the SD change:

(1) $$\mathrm{SD}\;\mathrm{change}=\;\sqrt{{\left([\mathrm{SD}\;\mathrm{pre}\right]}^{2}{+\;[\mathrm{SD}\;\mathrm{post}]}^{2}\;-\;2\;\times \;\mathrm{corr}\;-\;\left[\mathrm{pre},\mathrm{post}\right]\;\times \;\mathrm{SD}\;\mathrm{pre}\;\times \;\mathrm{SD}\;\mathrm{post})}$$

The correlation factor (f.corr) of 0.80 [32] was used for both the PEIC and sham/control groups. To estimate inter-study heterogeneity the I2 statistic was used, where I2 = 25% was considered low, I2 = 50% moderate and I2 = 75% high [33].

## 3. Results

The search, selection, and inclusion process revealed that only six articles met our inclusion criteria [6,7,24,27,28,29]. Of these six, four [6,7,24,29] presented favorable results to PEIC and performance recovery, and two studies [27,28] were judged not favorable to PEIC. No study showed a negative effect of PEIC on performance recovery. All studies not favorable to PEIC used cross-over design, while the studies favorable, just two implemented this design [24,29].

The quality scores of the analyzed studies achieved a mean of 12.3 ± 0.84 (81.7 ± 5.0%) points. Only one study obtained a value of 13.5 [6], other studies obtained scores 12.5 [28,29], 12.0 [24] and 11.5 [7,27] (Table S1).

There was a total of 106 participants (102 males and only four females) with an average age of 25.1 years (range of standard deviation of 1.0–7.0 years). Two studies involved healthy recreational trained subjects [7,24], one study was conducted in trained subjects [6], one in well-trained subjects [27], one in college level participants [28], and one in semi-professional soccer players [29]. 

The exercise protocols utilized to induce fatigue were different among studies but, in all of them, the authors prescribed only lower body exercises. Three studies performed the same exercises to evaluate performance and induce fatigue (i.e., the subjects performed maximal incremental test to induce fatigue and evaluated performance recovery) [6,7,24], and three included different types of exercise (e.g., one evaluated performance and the other fatigue) [27,28,29]. It is important to highlight that, in the study of Daab et al. [29], some exercises included in the protocol to evaluate performance (e.g., sprints) were also performed in the protocol to induce fatigue, which is specific for soccer. These results are presented in Table 2.

Table 3 shows information regarding PEIC procedures, interval between fatiguing exercise and PEIC intervention as well as the interval between PEIC application and performance test, information about possible PEIC effects and type of intervention (i.e., PEIC, sham or control). Only one study performed PEIC or sham intervention 5 min after effort [6] while the five others performed immediately after it [7,24,27,28,29]. At first glance, results do not apparently show a consensus on the PEIC procedure. Most of the studies included sham/placebo intervention but they did not include a control intervention. The study that included a control intervention did not present favorable responses to PEIC [27] and the authors did not include a sham intervention. No studies applied a remote PEIC, i.e., no studies utilized intermittent vascular occlusion applied to remote areas with respect to the exercise muscle group (e.g., applied the PEIC on the lower limbs and performed exercises with the upper limbs or the other way around).

Although it prevented the drop in exercise performance 24 h post fatigue protocol (Table 4), PEIC did not influence most of the physiological and perceptual variables (Table 5). The CK, a marker of tissue injury, was attenuated 24 h after the PEIC intervention in well-trained subjects to resistance exercise [7] and semi-professional soccer players [29], but CK was not attenuated in trained cyclists [6] and in academy rugby union players [28]. When we pooled data, the CK and MS percentage changes were higher in sham/control compared to PEIC intervention.

### Performance Recovery, Muscle Injury Markers and MS Analysis

**Figure 2:**
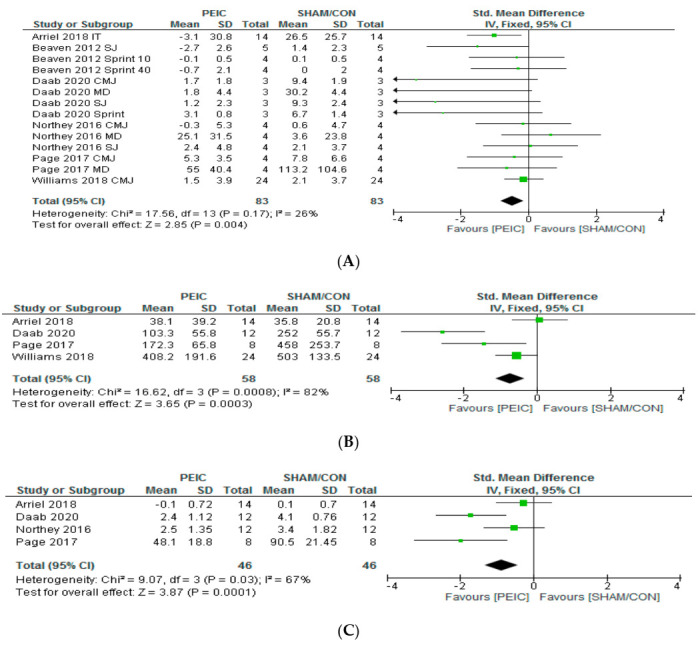
Forest plot of performance recovery (A) creatine kinase (B) and muscle soreness (C) variables between post-exercise ischemic conditioning (PEIC) and a cuff administration with low pressure (SHAM) or control (CON; no cuff) interventions. The square is the weight for a given study and is proportional to the weight of the study in the meta-analysis. The horizontal line indicates the 95% confidence interval (CI) for an effect. The diamond at the bottom represents the overall effect calculated using a fixed-effects model. IT = incremental test; SJ = squat jump; sprint 10 = 10 m sprint times over the 6 repeated sprints; sprint 40 = 40 m sprint times over the 6 repeated sprints; CMJ = countermovement jump; MD = muscle dynamometry; sprint = 20 m sprint.

The effect of PEIC on performance recovery, CK and MS is shown in Figure 2. Concerning performance recovery, there was a significant small effect size of PEIC compared to sham or control group (ES = −0.49, (CI: −0.82, −0.15), *p* = 0.004, Table S2). Moderate but non-significant heterogeneity was found amongst studies (I2 = 26%, *p* = 0.17). Regarding CK, there was a significant moderate effect size favoring PEIC compared to sham or control (ES = −0.74, (CI: −1.13, −0.34), *p* < 0.01, Table S3). A high significant heterogeneity was found amongst studies (I2 = 82%, *p* < 0.01). Finally, the increase in MS was significantly lowered by PEIC compared to sham or control, with a moderate effect size (ES = −0.88, (CI: −1.33, −0.43), *p* < 0.01, Table S4). A moderate significant heterogeneity was found amongst studies (I2 = 67%, *p* = 0.03).

## 4. Discussion

This is the first review and meta-analysis to analyze the effects and interpret the application of PEIC on the performance during recovery. Although a consensus among studies does not exist, our results indicate that PEIC leads to a significantly greater performance recovery and attenuates the CK increase 24 h post fatiguing protocols, especially in subjects with low to moderate levels of physical fitness. Among studies that did not present favorable results to PEIC, one did not include a placebo/sham intervention [27] and another did not include a control intervention [28] in their experimental design. In addition, both investigations adopted a cross-over design. These approaches could generate biased results because it was not possible to blind the subjects to PEIC intervention. Selected ones presented a high quality but only two described statistical significance in the measure of performance and physiological variables [7,29]. Considering that there is no standardization of the present nomenclature and that occlusion–reperfusion interventions are used indifferently prior or post exercise, we would like to suggest that in the future studies researchers use the term post-exercise ischemic conditioning (PEIC) when referring to this type of intervention.

### 4.1. Quality of the Papers

Although a high-quality score has been achieved by studies, some limitations were found. Most of the studies did not clearly describe the characteristics of the subjects included in the investigation (criterion 3) [7,27,28]. This can make it difficult to interpret results and to reapply the protocols used. Only two studies reported the exact *p*-value (criterion 10) for main results [28,29]. The latter one provided information about the strength of the evidence against the null hypothesis, avoiding doubts for the reader to make a decision. We have also concern about the appropriate use of statistical analysis (criterion 12) [7,24,28], as authors did not perform normality tests of data nor parametric tests for categorical variables (e.g., perceived exertion and pain perception). In addition, they utilized the independent test for paired sample. However, this inappropriate statistical did not influence our meta-analysis, since we have worked with raw data.

Finally, only one study included confidence intervals (CIs) for the main results (criterion 14) [24]. The CI is employed to show the dispersion or variability/reliability of an estimate, which likely includes the estimate of the average of populations. This is influenced by the sample size and the homogeneity of the data sample and it can be used to describe how reliable the results of a research study are. In addition, only two studies estimated the sample size and statistic power [6,29].

### 4.2. Participants Involved

Of 106 participants, only four were female, which reduces the possibility of suggesting the PEIC effects in women. The studies that demonstrated favorable effects of PEIC on performance recovery [6,7,24,29] involved healthy participants, semi-professional soccer players and recreationally trained or active subjects, while other studies involved well trained and college level participants [27,28]. There is no study with elite or high-trained athletes. This fact is comprehensible due to the difficulty in recruiting athletes available for this kind of research. Therefore, the positive effects of PEIC have not been investigated in this type of population because their physiological and performance responses are different from those found in non-athletes in several aspects. In addition, the responsiveness of IR intervention, when applied before exercise or test to improve performance, has been associated with the training level of the subjects. Specifically, it was demonstrated that participants with low fitness level presented large [35] while high training level small [19] or no response to this intervention [36]. In this context, since PEIC responses have the same pattern, it could be speculated that its responses are also dependent on the fitness level of subjects. However, it is important to highlight that, following the quality scores of studies, most of them did not clearly describe the characteristics of the enrolled subjects.

### 4.3. Exercise Protocols to Induce Fatigue and Assess Performance

The PEIC effects were primarily tested in exercise types that involved vertical jumps, sprints, and resistance exercise [24]. Although one study investigated used an incremental cycling test [6], the most common tests were jumps and maximal voluntary contraction [7,27,28,29]. Although these tests were largely used to evaluate changes in performance [37], they may not be sensitive enough to identify these changes 24 h after exercise [27]. Among studies selected for this review, some did not identify significant declines in exercise performance 24 h after the execution of fatiguing protocols in the sham/control, or PEIC groups when compared to pre-intervention. For example, in the Northey et al. [27] study, the performance of the countermovement jump and the concentric isokinetic peak torque measured in the pre-fatiguing protocol were not different after 24 h among and within groups. The same result was obtained by Williams et al. [28], who investigated peak power output and jump height. They found no difference 24 h after fatiguing protocol for both sham/control and PEIC groups when compared to pre-intervention. On the other hand, in the Arriel et al. [6] study, the sham group presented a drop of performance during incremental cycling 24 h after fatiguing protocol, but after PEIC this phenomenon was not observed. The same happened in the study by Page et al. [7] and by Daab et al. [29] but on the maximal isometric voluntary contraction. Alternatively, this fact may also mean that the physical exercise dose to induce fatigue was not able to cause a drop in performance 24 h after physical exercise. Therefore, these procedures may lead to misleading conclusions on the potential effects of PEIC 24 h post physical exercise.

### 4.4. PEIC Effects on Performance Recovery, CK and MS

Oxidative stress, muscular damage, increased inflammation, and MS associated with a decreased performance have all been reported after exhaustive exercise performed by different athletes involved in different sports [3,38,39], especially those over the course of the multi-day races (i.e., 2 to 7 consecutive days of competition) [3,39] and with high training volumes [40]. This phenomenon may be useful for athletes and coaches searching strategies that minimize decrements in performance or to accelerate the recovery from fatiguing efforts. As exposed in Figure 2, our statistical analysis showed a favorable PEIC effect on performance recovery, CK (muscle damage markers), and MS. Although there is not enough evidence to support a remarkable use of the PEIC immediately after exercise to reduce these biomarkers and to speed recovery, this intervention could lead to an improvement in recovery in these individuals.

It is important to note that all studies analyzed by the present review evaluated only the acute effect of PEIC application. Although there is no study that investigated the repeated effect of PEIC application (i.e., several days of application), recent study showed that several days of application of blood flow restriction (just one cycle, and lower pressure than PEIC intervention) after resistance exercise was associated with an impaired muscle adaptation [41], and this fact may be due to magnitude of oxidative stress. The oxidative stress, when moderate, plays multiple regulatory roles in cells, such as regulation of cell signaling pathways. However, it was speculated that low-to-none levels are not beneficial [42]. Therefore, as cycles of IR were associated with the attenuated stress oxidative level [43,44], we suggest that PEIC application should be performed before main competitions or when athletes incorporate a high training volume session. However, further studies are necessary to investigate the PEIC contribution when applied repeatedly on different exercise modes and kinds of sport activities.

### 4.5. PEIC Protocols and Possible Mechanism

At first glance, there is no consensus among researchers about the number and duration of IR cycles during PEIC. Furthermore, no consensus exists on the period between PEIC application and the return to exercise and whether participants involved were informed about the possible effects of PEIC.

The cycles of ischemia presented a total time ranging from 6 to 15 min and the complete PEIC protocol (occlusion and reperfusion periods) from 12 to 30 min (Table 3). Only one study analyzed two different protocols [6]. While some authors using a total time of ischemia above 10 min described positive effects on recovery [7,29,35], others using shorter time did not [27,28]. Although Beaven et al. [24] found positive results for PEIC using total time of 6 min, in this investigation subjects were recreational athletes. Only one study verified two different PEIC protocols with the same total time of ischemia (10 min), but authors did not find significant difference between protocols [6]. A clinical study [45] that demonstrated cardiac injury protection applying intermittent vascular occlusion before prolonged ischemic insult, reported that 4 to 6 ischemic cycles lasting 2 to 5 min yielded significant cardioprotection. Therefore, in addition to training level, it is conceivable that a minimal dose of ischemia is necessary to generate a PEIC response and that highly trained subjects would need a higher dose of PEIC than trained and untrained individuals.

The time between PEIC application and the return to exercise was usually 24 h. However, PEIC effects were also evaluated at 5 min, 1 h, 2 h, 48 h and 72 h. While no positive effects were found between 1 and 2 h after PEIC application [27,28], 24 h has revealed significant changes [6,7,24,29]. Among the few established IR intervention mechanisms, the early (active immediately after reperfusion and lasts 2–3 h) and late (begins 12–24 h after reperfusion) phases of protection, commonly known as the first and second window of protection [16], should be considered. Looking at our results, the second phase appears to be more pronounced. However, some variables (e.g., CK and MS) used to assess the effects of PEIC on recovery have their peak 24 h post exercise. Therefore, it remains unclear whether the first, the second or both phases are effective on performance recovery.

Finally, we can hypothesize that on the early phase, the PEIC could increase nitric oxide and modulate mitochondrial oxygen consumption leading to a decrease in mitochondrial reactive oxygen species (ROS) generation [43] and consequently limit oxidative injury on muscle cell. During the late phase, PEIC could increases iNOS expression (an isoform that synthesizes nitric oxide) [46], and consequently increases nitric oxide production, leading to a possible improvement in exercise performance by diminishing the level of ROS [43]. These occurrences are also associated with reduced muscle fatigue and damage [47]. In addition, it was speculated that PEIC could lead to an attenuated inflammatory response after physical exercise due to a downregulation of circulating leukocytes [7]. This could be, at least in part, responsible for the beneficial effects of PEIC on recovery.

## 5. Conclusions

PEIC intervention has proved to be an effective, non-invasive, inexpensive and easy-to-apply strategy to accelerate performance recovery by the attenuation of creatine kinase and muscle soreness increase in subjects with a low-to-moderate fitness level. In highly trained subjects, a higher dose of PEIC may be administered to elucidate beneficial effects, while the ideal PEIC protocol has not been standardized yet. This intervention is a new approach in the field of ischemic conditioning, which can be applied after exercises that potentially cause muscle damage and soreness such as during multi-day competitions or high training volumes sessions.

## Figures and Tables

**Table 1:** Checklist used to analyze the quality of publications.

		Points	
Reporting	0	½	1
1. Is the hypothesis/aim/objective of the study clearly described?	No	Unclear	Yes
2. Are the main outcomes to be measured clearly described in the Introduction?	No	Unclear	Yes
3. Are the characteristics of the subjects included in the study clearly described?	No	Unclear	Yes
4. Are the interventions of interest clearly described?	No	Unclear	Yes
5. Are the main findings of the study clearly described?	No	Unclear	Yes
6. Does the study provide estimates of the random variability in the data for the main outcomes?	No	Unclear	Yes
7. Were the instruments of testing reliable?	No	Unclear	Yes
8. Was a follow-up duration sufficiently described and consistent within the study?	No	Unclear	Yes
9. Number of participants included in study findings	<5	6–15	>16
**Analysis and presentation**			
10. Have actual probability values been reported (e.g., 0.035 rather than <0.05) for the main outcomes except, where the probability value is less than 0.001?	No	Unclear	Yes
11. Was there a statement adequately describing or referencing all statistical procedures used?	No	Unclear	Yes
12. Were the statistical analyses used appropriate?	No	Unclear	Yes
13. Was the presentation of results satisfactory?	No	Unclear	Yes
14. Were confidence intervals given for the main results?	No	Unclear	Yes
15. Was the conclusion drawn from the statistical analysis justified?	No	Unclear	Yes

**Table 2:** Characteristics of the PEIC studies.

	N	Male	Female	Samples	Subjects	(Exercise) Fatigue Protocol	Exercise/Test to Assess Performance	Is PEIC Favorable to Performance?	Other Variables Analyzed
Beaven et al. (2012) [24]	14	10	4	Paired	Healthy Recreationally trained	Jumps/sprints/leg press test	Jumps/sprints/leg press test	Yes	#
Northey et al. (2016) [27]	12	12	0	Paired	Healthy well trained (resistance exercise)	Back Squat (10 sets × 10 repetitions (70% 1RM))	MVC/Jumps	No	MS and PRS
Page et al. (2017) [7]	16	16	0	No Paired	Healthy Recreationally active	Jumps (5 sets × 20 repetitions own body weight (box 0.6 m height))	MIVC/Jumps	Yes	MS *, CK *, TC
Williams et al. (2018) [28]	24	24	0	Paired	Rugby Player (college level)	6 sets × 50 m sprints	Jumps	No	MS, PRS, CK, lactate, cortisol and testosterone
Arriel et al. (2018) [6]	28	28	0	No Paired	Trained cyclists	Maximal Incremental	Maximal Incremental	Yes	MS, PRS, RPE, CK, HR
Daab et al. (2020) [29]	12	12	0	Paired	Semi-professional soccer players	Loughborough intermittent shuttle test ^&^	Jumps/Sprint/MVC	Yes	MS *, CK *, LDH *, CRP

PEIC, post-exercise ischemic conditioning; MS, muscle soreness; PRS, perceived recovery; RPE, perceived exertion; TC, thigh circumference; CK, creatine kinase; heart rate, HR; LDH, lactate dehydrogenase; CRP, serum C-reactive protein. ^&^, protocol designed to simulates the activities of real soccer match (six exercise sets lasting approximately 15 min (between 55% and 95% VO2 Max) separated by periods of 3 min) [34]; #, no evaluated. * It was influenced by PEIC.

**Table 3:** Characteristics of the PEIC protocols.

	PEIC Sets	Total PEIC and SHAM Time (min)	Ischemia Pressure (mm Hg) PEIC/SHAM/Limb	Time to Test	Groups	Were Subjects Informed about Effects of PEIC?
Beaven et al. (2012) [24]	2 × 3 min	6	220/15/thigh	5 min–24 h	PEIC/SHAM	No
Northey et al. (2016) [27]	2 × 3 min	6	220/#/thigh	1–24 h	PEIC/CON	It was not exposed by authors
Page et al. (2017) [7]	3 × 5 min	15	220/20/thigh	24–48–72 h	PEIC/SHAM	No
Williams et al. (2018) [28]	2 × 3 min	6	171−266/15/thigh	2–24 h	PEIC/SHAM	Yes
Arriel et al. (2018) [6]	2 × 5 min and 5 × 2 min	10 and 10	50 > SAP/20/thigh	24 h	PEIC/SHAM	Yes
Daab et al. (2020) [29]	3 × 5 min	15	50 > SAP/20/thigh	0–24–48–72 h	PEIC/SHAM	It was not exposed by authors

PEIC, post-exercise ischemic conditioning; SHAM, cuff administration with low pressure; CON, control (no cuff); SAP, systolic arterial pressure; #, no SHAM application.

**Table 4:** Comparison of the performance recovery between PEIC and SHAM/CON.

KERRYPNX		PEIC			SHAM/CON		
	Exercise	Pre-Intervention	24-h Post-Intervention	Change (%)	Pre-Intervention	24-h Post-Intervention	Change (%)
Beaven et al. (2012) [24]	SJea (m.s^−2^)	20.1 ± 3.9	22.8 ± 4.3	13.4	18.9 ± 3.7	17.5 ± 3.6	−7.4
	S 10 m (s)	12.5 ± 0.8	12.4 ± 0.8	0.8	12.6 ± 0.7	12.7 ± 0.8	−0.8
	S 40 m (s)	42.5 ± 3.4	41.8 ± 3.3	1.7	42.7 ± 3.1	42.7 ± 3.2	0.0
Northey et al. (2016) [27]	CMJ (cm)	41.8 ± 8.8	42.1 ± 6.9	0.7	42.7 ± 7.7	42.1 ± 7.0	−1.4
	SJ (cm)	37.7 ± 7.8	35.3 ± 7.2	−6.4	38.1 ± 5.6	36.0 ± 6.1	−5.5
	MD (30 deg.s^−1^) (Nm)	281.5 ± 46.0	256.4 ± 52.0	−8.9	273.7 ± 35.5	270.1 ± 39.1	−1.3
Page et al. (2017) [7]	CMJ (cm)	34.0 ± 4.4	28.7 ± 1.2	−15.6	38.9 ± 8.1	31.1 ± 2.0	−20.1
	MD (N)	611.0 ± 51.0	556.0 ± 67.2	−9.0	629.0 ± 136.0	515.8 ± 43.3	−18.0
Williams et al. (2018) [28]	CMJ (cm)	40.4 ± 6.0	38.9 ± 6.2	−3.7	39.7 ± 6.0	37.6 ± 5.6	−5.3
Arriel et al. (2018) [6]	IT (s)	808.3 ± 122.9	811.4 ± 135.1	0.4	779.9 ± 122.9	753.4 ± 110.0	−3.4
Daab et al. (2020) [29]	CMJ (%)	100.0 ± 0.0	98.3 ± 1.8	−1.7	100.0 ± 0.0	90.6 ± 1.9	−9.4
	SJ (%)	100.0 ± 0.0	98.8 ± 2.3	−1.2	100.0 ± 0.0	90.7 ± 2.4	−9.3
	MD (%)	100.0 ± 0.0	98.2 ± 4.4	−1.8	100.0 ± 0.0	69.8 ± 4.4	−30.2
	S 20 m (%)	100.0 ± 0.0	103.1 ± 0.8	−3.1	100.0 ± 0.0	106.7 ± 1.4	−6.7
Mean				−2.5 *			−8.5

PEIC, post-exercise ischemic conditioning; SHAM, cuff administration with low pressure; CON, control (no cuff); SJea = squat jump eccentric acceleration; S 10 m = 10 m sprint times over the 6 repeated sprints sprint of 10 m; S 20 m = 20 m sprint; S 40 m = 40 m sprint times over the 6 repeated sprints; MD = muscle dynamometry; IT = incremental test; CMJ = countermovement jump; SJ = squat jump. *, different from SHAM/CON, *p* = 0.048.

**Table 5:** Results of the perceived recovery (PRS), muscle soreness (MS) and creatine kinase (CK) between PEIC and SHAM/CON.

		PEIC			SHAM/CON		
	Exercise	Pre-Intervention	24-h Post-Intervention	Change (%)	Pre-Intervention	24-h Post-Intervention	Change (%)
Northey et al. (2016) [27]	PRS (scores)	8.1 ± 1.5	5.6 ± 1.6	−30.9	7.9 ± 0.9	5.1 ± 1.9	−35.4
	MS (scores)	0.6 ± 0.8	3.1 ± 1.9	416.7	1.0 ± 0.8	4.4 ± 2.4	340.0
Page et al. (2017) [7]	MS (scores)	8.9 ± 8.0	57.0 ± 24.6	540.5	15.6 ± 12.5	106.1 ± 30.1	580.1
	CK (U/L)	163.5 ± 30.1	335.8 ± 87.3	105.4	178.4 ± 61.4	636.4 ± 300.1	256.7
Williams et al. (2018) [28]	CK (U/L)	218.9 ± 81.9	627.1 ± 250.7	186.5	228.6 ± 81.9	731.6 ± 189.7	220.0
Arriel et al. (2018) [6]	PRS (scores)	8.2 ± 2.2	7.2 ± 2.0	−12.2	7.5 ± 2.3	7.4 ± 1.7	−1.3
	MS (scores)	0.8 ± 1.2	0.7 ± 0.9	−12.5	0.6 ± 1.1	0.7 ± 1.1	16.7
	CK (U/L)	205.9 ± 138.4	244.0 ± 160.2	18.5	192.5 ± 127.6	228.3 ± 138.5	18.6
Daab et al. (2020) [29]	MS (scores)	0.5 ± 0.1	2.9 ± 1.2	480.0	0.6 ± 0.5	4.7 ± 1.1	683
	CK (%)	100.0 ± 0.0	203.3 ± 55.8	103.3	100.0 ± 0.0	352.0 ± 55.7	252.0
Mean	PRS			−21.6			−18.4
	MS			356.2			404.9
	CK			103.4			186.8

PEIC, post-exercise ischemic conditioning; SHAM, cuff administration with low pressure; CON, control (no cuff).

## Supplementary Materials

The following are available online at https://www.mdpi.com/1660-4601/17/21/8161/s1, Table S1: Scores assigned to each of the studies for each of the quality (Q) criteria, Table S2: Complementary data from the forest plot (Figure 2) of performance recovery, Table S3: Complementary data from the forest plot (Figure 2) of creatine kinase, Table S4: Complementary data from the forest plot (Figure 2) of muscle soreness.
